# Cirrhosis in Wilson Disease is characterized by Impaired Hepatic Synthesis, Leukopenia and Thrombocytopenia

**DOI:** 10.7150/ijms.44338

**Published:** 2020-05-29

**Authors:** Hao-Jie Zhong, Ping Xiao, Da Lin, Hui-Min Zhou, Xing-Xiang He

**Affiliations:** 1 Guangdong Medical University, Zhanjiang, China.; 2 Department of Gastroenterology, The First Affiliated Hospital of Guangdong Pharmaceutical University, Guangzhou, China.; 3 Department of Gastroenterology, Jieyang People's Hospital, Jieyang, China.

**Keywords:** Wilson disease, Cirrhosis, Liver dysfunction, Portal hypertension, Splenomegaly

## Abstract

**Background:** Patients with Wilson disease (WD) progress to cirrhosis at an early age but have good prognoses. This study aimed to delineate hepatic features in WD patients with or without cirrhosis.

**Methods:** Medical data were retrospectively collected from 27 July 2015 to 27 June 2018. WD patients were divided into two groups based on whether or not they progressed to cirrhosis. Liver function, portal hypertension features and hematocytopenia rates were compared between groups.

**Results:** The study enrolled 119 WD patients with cirrhosis and 53 WD patients without cirrhosis. There were no differences between groups for liver enzyme levels or incidence rates of Kayser-Fleischer ring (all *P* > 0.05). Ascites and hepatic encephalopathy were nearly absent in both groups, and almost all patients were Child-Pugh group A. However, WD-associated cirrhotic patients had a higher prothrombin time (beta = 0.908, *P* = 0.004) and international normalized ratio (beta = 0.089, *P* = 0.040), wider portal vein diameter (beta = 1.330, *P* < 0.001), and an increased risk of splenomegaly/splenectomy (odds ratio [OR] = 4.36, 95% confidence interval [CI]: 2.15-8.84, *P* < 0.001). Moreover, WD-associated cirrhotic patients have significantly increased risks of leukopenia (OR = 2.30, 95% CI: 1.00-5.25, *P* = 0.049) and thrombocytopenia (OR = 6.89, 95% CI: 2.01-23.59, *P* = 0.002).

**Conclusions:** Despite presenting good outcomes, mild hepatocyte injury, and good hepatic metabolic function, WD-associated cirrhotic patients show more serious impairment of hepatic synthetic function, wider portal vein diameter, and higher risk of splenomegaly due to portal hypertension.

## Introduction

Cirrhosis is the eleventh-most common cause of death globally and is characterized by hepatic dysfunction, portal hypertension, and high risk of hepatocellular carcinoma (HCC) 1,2. Compared with those with liver disease and no cirrhosis, cirrhotic patients often showed more severe hepatocyte injury; worse declines in hepatic synthetic function; and higher prevalence rates of variceal bleeding, encephalopathy, and splenomegaly, especially among cases caused by non-alcoholic fatty liver disease and viral hepatitis 3-5. It is these manifestations and complications, rather than the presence of comorbidities, which should account for the increased mortality of cirrhotic patients 1,6. Therefore, it is of great importance to clarify the hepatic features in patients with or without cirrhosis.

Wilson disease (WD) is an autosomal recessive disorder of copper metabolism, caused by mutations in the *ATP7B* gene that encodes a copper-transporting P-type ATPase 7. Unlike most cases of cirrhosis attributable to other causes, patients with WD frequently progress to cirrhosis at a young age, one-third while still children or adolescents 8,9. Nevertheless, the prognosis of WD-associated cirrhosis is excellent, and appropriate treatment can normalize many laboratory test results 10. Long-term follow-up studies found no increased risk of HCC (0.14%/year) in WD-associated cirrhotic patients 11,12. Interestingly, the hepatic features of WD-associated cirrhosis are very distinct from those of hepatitis B-associated cirrhosis. Patients present better liver function but a higher risk of splenomegaly, suggesting that cirrhosis is not a single disease entity 13. Therefore, it is crucial to characterize the hepatic features in WD patients with or without cirrhosis to guide further treatment and prevent complications. Thus, we performed this study to delineate the hepatic features in WD patients with or without cirrhosis.

## Methods

### Study design and participants

This cross-sectional study was performed at the First Affiliated Hospital of Guangdong Pharmaceutical University, Guangzhou, China. The subjects were inpatients who had been diagnosed with WD at this hospital from 27 July 2015 to 27 June 2018. The patients were excluded if they met any of the following criteria: (1) carcinoma; (2) severe heart or pulmonary diseases; (3) other hepatic conditions such as alcoholic, fatty, viral, autoimmune, drug-induced, or parasitic liver disease; or (4) important medical data missing. Each patient was recorded only once.

This study was performed with the approval of the First Affiliated hospital of Guangdong Pharmaceutical University Institutional Review Board (No. 2019-60) and adhered to the Declaration of Helsinki. Given that this was a retrospective study, the Institutional Review Board waived the requirement for informed consent from each patient.

### Data collection

We reviewed medical data of all enrolled subjects to collect the information on gender, age at time of data collection, body mass index (BMI); history of alcohol consumption and smoking; underlying diseases (diabetes and hypertension); duration and manifestations of WD; imaging findings; and laboratory test results including alanine transaminase (ALT), aspartate transaminase (AST), serum albumin, prothrombin time (PT), international normalized ratio (INR), total cholesterol, triglyceride, bilirubin, white blood cell (WBC) count, red blood cell (RBC) count, and platelet count.

Alcohol use was defined as the alcohol consumption >140 g per week. Hypoalbuminemia was defined as serum albumin <30 g/L. Hyperbilirubinemia was defined as bilirubin >34 μmol/L. Leukopenia was diagnosed in patients with WBC counts <4.0 × 10^9^/L. Erythropenia was defined as RBC count <4.0 × 10^12^/L (male) or <3.5 × 10^12^/L (female). Thrombocytopenia was diagnosed at platelet counts <100 × 10^9^/L. In China, cirrhosis was diagnosed based on liver biopsy or combination of clinical manifestations (hepatomegaly, splenomegaly, ascites, hepatic encephalopathy and others), laboratory examinations (ALT, AST, procollagen type III N-terminal propeptide, type IV collagen, laminin, hyaluronic acid and others), and imaging examinations (typical morphological changes, including a blunted, nodular liver edge accompanied by splenomegaly in abdominal ultrasonography or computed tomography).

### Clinical diagnosis of WD

In China, WD is diagnosed based on a combination of clinical features and laboratory parameters: onset age, family history, symptoms, serum ceruloplasmin <200 mg/L, urinary copper excretion ≥100 μg/24 h; liver copper >250 μg/g dry weight; urinary copper excretion >1600 μg/24 h after the administration of 2 × 500 mg D-penicillamine; presence of Kayser-Fleischer ring by slit-lamp examination; and mutation analysis 14. Although the Chinese diagnosis criteria were not based on a score system, all the tests in the international criteria were included in these criteria 15.

### Statistical analysis

Statistical analyses were performed using SPSS software, version 22 (IBM Corp., Armonk, NY, USA). Results are presented as frequencies and proportions for categorical variables, means ± standard deviation for normally distributed continuous variables, and medians and interquartile ranges for non-normally distributed continuous variables. Chi-square or Fisher exact tests were used for group comparisons of categorical variables. Student *t*-tests were performed for group comparisons of normally distributed data, and Mann-Whitney *U*-tests were applied for non-normally distributed data. To assess the association between cirrhosis and laboratory results in WD patients, multivariable linear regression analysis with a stepwise method was used to adjust for confounders (*i.e.* age, sex, body mass index, disease duration, hypertension, diabetes, alcohol use, and smoking). Logistic regression analysis with a backward conditional method was carried out to assess the relationship of hepatic features with cirrhosis. The results are reported as odds ratios (ORs) and 95% confidence intervals (CIs). Two-sided *P*-values < 0.05 were considered statistically significant.

## Results

### Patient characteristics

After excluding six patients with carcinoma and seven patients with severe heart or pulmonary diseases, 119 WD patients with cirrhosis and 53 WD patients without cirrhosis were enrolled. The clinical characteristics of the two groups are presented in **Table 1**. Most of the diagnoses of cirrhosis (103/119) were made based on patients' medical history. Sixteen patients were newly diagnosed cirrhosis. All enrolled patients were undergoing copper lowering treatment for WD.

### Liver function features in WD patients with or without cirrhosis

WD patients with cirrhosis had significantly lower levels of albumin and triglyceride, while PT, INR, and bilirubin level were significantly higher compared to WD patients without cirrhosis (*P* < 0.05, **Table 2**). The total cholesterol level was higher in WD-associated cirrhotic patients, but the difference was not significant (**Table 2**). There were no significant between-group differences for ALT and AST levels, and incidence rates of Kayser-Fleischer ring. Hepatic encephalopathy was almost absent in both groups, and almost all patients were in Child-Pugh group A.

After adjustment for confounders, WD-associated cirrhotic patients still showed higher PT and INR and lower levels of total cholesterol and triglyceride; there were no differences for other liver function indices compared with patients with WD without cirrhosis (**Tables 3 and 4**).

### Portal hypertension features in WD patients with or without cirrhosis

The portal vein diameter and prevalence of splenomegaly (or splenectomy) were significantly higher in WD-associated cirrhotic patients (**Table 2**). And ascites was almost absent in both groups.

Multivariable linear regression after confounder adjustment showed that WD-associated cirrhotic patients had a wider portal vein diameter (beta = 1.330, *P* < 0.001, **Table 3**). Moreover, logistic regression analysis showed that WD-associated cirrhotic patients also had a significantly increased risk of splenomegaly (or splenectomy) compared with non-cirrhotic patients (OR = 4.36, 95% CI: 2.15-8.84, *P* < 0.001, **Table 4**).

### Hematocytopenia in WD patients with or without cirrhosis

Compared with those without cirrhosis, WBC, RBC and platelet counts were significantly decreased in WD-associated cirrhotic patients, both before and after adjustment for confounders (**Tables 2 and 3**). The incidence of leukopenia (33.05% vs. 16.98%, *P* = 0.031) and thrombocytopenia (29.66% vs. 5.66%, *P* < 0.001) were also higher in WD patients with cirrhosis (**Table 2**). After adjustment, patients with WD-associated cirrhosis showed significantly increased risks of leukopenia (OR = 2.30, 95% CI: 1.00-5.25, *P* = 0.049) and thrombocytopenia (OR = 6.89, 95% CI: 2.01-23.59, *P* = 0.002; **Table 4**).

Seventy-six patients with WD-associated cirrhosis had splenomegaly; 8 underwent partial splenectomy, 12 had complete splenectomy, and 56 did not undergo splenectomy (included 1 patient with no routine blood test results). Only 8.33% (1/12) patients with complete splenectomy had leukopenia and 0% (0/12) had thrombocytopenia, which were much lower rates compared to patients who underwent partial splenectomy (87.50% and 62.50%) or did not have surgery (45.45% and 45.45%, **Table 5**).

## Discussion

Cirrhosis, as an end-stage liver disease, leads to liver dysfunction and portal hypertension that can cause fatal complications such as liver failure, variceal bleeding, hepatic encephalopathy, severe infections, and even death 2,16. Mounting evidence suggests that cirrhosis should no longer be considered a single disease, because patients with cirrhosis due to different causes may present with variable clinical features 2,13. WD patients progress to cirrhosis at an early age but have good prognosis, which might contribute to its distinct hepatic features. However, the clinical features that distinguish WD patients with and without cirrhosis are still unclear. To the best of our knowledge, this is the first study to compare liver features in these two groups of WD patients. The findings might help facilitate early diagnosis and prompt treatment to prevent complications when WD patients progress to cirrhosis.

As a comprehensive index to evaluate liver functional reserve, the Child-Pugh classification is widely used to predict the prognosis of cirrhotic patients 17. We found that almost all WD-cirrhosis patients were in Child-Pugh group A, which predicted good outcomes in WD-associated cirrhosis patients, similar with previous studies 7,10. Cirrhotic patients typically present more with serious hepatocyte injury compared with non-cirrhotic patients 4,18. However, we found no difference in hepatocyte injury (based on ALT and AST) between WD patients with and without cirrhosis. Consistent with this, previous studies showed that aminotransferase levels could decrease and reach nearly normal values in WD patients who undergone copper lowering treatment, despite the continued presence of cirrhosis 19,20. A long-term follow-up study assessed liver fibrosis in WD patients by biopsy and found marked reductions of liver fibrosis in WD-associated cirrhotic patients on copper removal treatment 21. Their results demonstrated that advanced fibrosis could reverse in controlled WD. This suggests that the prognosis for WD-associated cirrhotic patients might improve with appropriate treatment.

The liver plays a crucial role in synthesizing albumin, coagulation factors, and lipids and is involved in ammonia and bilirubin metabolism 22. As a feature of cirrhosis, liver dysfunction was assessed based on the synthesis and metabolism of these substances. The incidences of hypoalbuminemia (5.04%), hyperbilirubinemia (4.20%), and hepatic encephalopathy (0.84%) were low in WD-associated cirrhotic patients. Moreover, multivariable linear and logistic regression analyses showed no differences in albumin level, bilirubin level between the two groups. These findings suggest that WD patients seem to have good liver metabolic functions despite the presence of cirrhosis. However, compared with those without cirrhosis, WD patients with cirrhosis presented higher PT and INR and lower total cholesterol and triglyceride levels, which indicated they have impaired synthesis of coagulation factors and lipids.

As another feature of cirrhosis, portal hypertension was assessed based on the presence of ascites, portal vein diameter, and splenomegaly. Ascites was almost absent in both groups, and cirrhosis was not associated with a higher risk of ascites in WD patients. This might be due to the high concentration of serum albumin in cirrhotic patients, which might result in high plasma osmotic pressure and reduce the occurrence of ascites 23. Nevertheless, compared with those without cirrhosis, WD-associated cirrhotic patients had a wider portal vein diameter. This indicates that these patients might have higher portal vein pressure that could predispose them to esophageal varices 24.

In normal physiology, splenic blood flows back to the liver through the portal vein. In cirrhosis, spleen congestion secondary to portal outflow resistance leads to splenomegaly, which is an important clinical indicator of portal hypertension 25. A previous report described the clinical features of 910 cirrhotic patients showed that about half (50.50%) presented with splenomegaly 26. The present study found that the incidence of splenomegaly in WD-associated cirrhotic patients was higher than that in non-cirrhotic patients (63.87% vs. 28.30%) and also higher than in the previous study (50.50%) 26. After adjustment for potential confounders, WD-associated cirrhotic patients showed a 4.36-fold risk of splenomegaly compared to non-cirrhotic patients.

Splenomegaly in WD patients frequently causes functional overactivity of the spleen (known as hypersplenism), and some anti-copper medications such as penicillamine can occasionally cause bone marrow depression, anemia, and leukopenia 27,28. Therefore, we also assessed hematocytopenia rates in both groups 29. We found that patients with WD-associated cirrhosis showed reduced WBC, RBC, and platelet counts and increased risks of leukopenia and thrombocytopenia, which might increase their risks of serious infection and spontaneous bleeding 27. A previous study demonstrated that infections increase mortality by four-fold in patients with cirrhosis; 30% die within a month after infection and another 30% die by 1 year, and variceal bleeding and intracerebral bleeding due to thrombocytopenia can also be fatal 30. Thus, we should closely evaluate WD patients for leukopenia and thrombocytopenia when they progress to cirrhosis.

A study from China included 65 patients with splenomegaly secondary to cirrhosis and showed improvements in WBC and platelet counts after splenectomy 31. Additionally, another study of 70 WD patients with splenomegaly reported a similar result 27. Consistent with these results, we found that the incidence rates of leukopenia and thrombocytopenia were significantly reduced in WD patients who underwent complete splenectomy to treat splenomegaly secondary to cirrhosis. This finding revealed that complete splenectomy was a useful treatment for leukopenia and thrombocytopenia in WD-associated cirrhotic patients, and it was more efficient than partial splenectomy.

There are several limitations of this study. First, there was the possibility of selection bias, since we only enrolled in-patients who might have more severe WD and more comorbid diseases compared with outpatients. Second, some hepatic complications such as esophageal varices, hepatorenal syndrome, and hepatocellular carcinoma were hard to assess due to a lack of clinical data. Third, to explore the effect of splenectomy on treatment of hematocytopenia, to compare the data of patients before and after splenectomy was the most ideal way. However, due to the lack of data before splenectomy, we only compared the data among with complete splenectomy, partial splenectomy and non-splenectomy. Fourth, although WD is rare, the sample size of our study was still small. These results should be interpreted with caution, and further confirmatory studies are needed.

## Conclusions

In conclusion, despite good outcomes, mild hepatocyte injury, and good hepatic metabolic function, patients with WD-associated cirrhotic showed more serious impairment of hepatic synthetic function, wider portal vein diameter, and higher risk of splenomegaly due to portal hypertension. More attention should be paid to the insufficiency of coagulation factors due to hepatic synthetic dysfunction and leukopenia and thrombocytopenia due to hypersplenism; addressing these areas could lower the risks of infection and bleeding. Complete splenectomy might be an effective therapeutic measure for leukopenia and thrombocytopenia in WD-associated cirrhotic patients.

## Figures and Tables

**Table 1:** Patient characteristics

	Cirrhosis (n = 119)	Non-cirrhosis (n = 53)	*P* -value
Age (yr)	25.00 (21.00-33.00)	21.00 (14.50-29.00)	0.003
Male sex	56 (47.06)	29 (54.72)	0.354
Body mass index (kg/m^2^)	19.61 (17.98-21.78)	18.85 (16.72-20.64)	0.016
Disease duration (years)	(n = 119)	(n = 52)	0.971
**<1**0	77 (64.71)	33 (63.46)	
10-19	34 (28.57)	15 (28.85)	
≥20	8 (6.72)	4 (7.69)	
Diabetes	1 (0.84)	0 (0)	1.000
Hypertension	1 (0.84)	0 (0)	1.000
Alcoholism	1 (0.84)	1 (1.89)	0.523
Smoking	12 (10.08)	3 (5.66)	0.511
Neurological manifestations	85 (71.43)	27 (50.94)	0.009
Psychiatric manifestations	6 (5.04)	0 (0)	0.179

Data are presented as median (interquartile range) or n (%).

**Table 2:** Hepatic features in WD patients with or without cirrhosis

	Cirrhosis (n = 119)	Non-cirrhosis (n = 53)	*P*-value
Liver function features			
ALT (U/L)	22.00 (15.00-34.00)	23.00 (14.00-55.50)	0.508
AST (U/L)	23.00 (18.00-38.00)	23.00 (18.00-38.90)	0.797
Albumin (g/L)	39.00 (36.00-41.10)	40.00 (36.65-44.00)	0.044
PT (s)	14.40 (13.60-15.30)	13.50 (12.95-14.25)	<0.001
INR	1.12 (1.04-1.21)	1.03 (0.97-1.10)	<0.001
Total cholesterol (mmol/L)	3.67 (3.06-4.45) (n = 88)	3.98 (3.65-4.66) (n = 40)	0.054
Triglyceride (mmol/L)	0.82 (0.62-1.06) (n = 88)	0.98 (0.81-1.32) (n = 40)	0.010
Bilirubin (μmol/L)	13.30 (9.10-18.10)	11.10 (7.75-13.75)	0.010
Hepatic encephalopathy	1 (0.84)	0 (0)	1.000
Kayser-Fleischer ring	39 (32.77)	13 (24.53)	0.277
Child-Pugh classification			0.225
A	113 (94.96)	53 (100)	
B/C	6 (5.04)	0 (0)	
Portal hypertension features			
Portal vein diameter (mm)	10.00 (10.00-11.00) (n = 88)	9.00 (8.00-10.00) (n = 40)	<0.001
Ascites	3 (2.52)	1 (1.89)	1.000
Splenomegaly/splenectomy	76 (63.87)	15 (28.30)	<0.001
Hematocytopenia			
WBC (10^9^/L)	4.60 (3.38-5.75)	5.04 (4.43-6.50)	0.012
RBC (10^12^/L)	4.37 (3.93-4.83)	4.61 (4.14-4.96)	0.021
Platelet (10^9^/L)	170.96 ± 89.59	227.49 ± 93.00	<0.001
Leukopenia	39 (33.05)	9 (16.98)	0.031
Erythropenia	16 (13.56)	5 (9.43)	0.447
Thrombocytopenia	35 (29.66)	3 (5.66)	<0.001

Data are presented as means ± standard deviation, medians (interquartile ranges) or n (%). ALT, alanine transaminase; AST, aspartate transaminase; INR, international normalized ration; PT, prothrombin time; RBC, red blood cell; WBC, white blood cell; WD, Wilson disease.

**Table 3:** Multivariable linear regression analysis of hepatic features in WD patients with and without cirrhosis

	Beta	Standard error	*P*-value
Liver function indices			
ALT (U/L)	-	-	-
AST (U/L)	-	-	-
Albumin (g/L)	-	-	-
PT (s)	0.908	0.314	0.004
INR	0.089	0.032	0.040
Total cholesterol (mmol/L)	-0.400	0.199	0.047
Triglyceride (mmol/L)	-0.299	0.145	0.041
Bilirubin (μmol/L)	-	-	-
Portal hypertension			
Portal vein diameter (mm)	1.330	0.320	<0.001
Hematocytopenia			
WBC (10^9^/L)	-0.709	0.286	0.014
RBC (10^12^/L)	-0.253	0.127	0.048
Platelet (10^9^/L)	-71.016	13.615	<0.001

Adjusted for age, sex, body mass index, disease duration, hypertension, diabetes, alcoholism and smoking. ALT, alanine transaminase; AST, aspartate transaminase; INR, international normalized ration; PT, prothrombin time; RBC, red blood cell; WBC, white blood cell; WD, Wilson disease.

**Table 4:** Logistic regression analysis of hepatic features in WD patients with and without cirrhosis

	OR	95% CI	*P*-value
Liver function indices			
Hepatic encephalopathy	-	-	-
Kayser-Fleischer ring	-	-	-
Child-Pugh classification	-	-	-
Portal hypertension			
Ascites	-	-	-
Splenomegaly/splenectomy	4.36	2.15-8.84	˂0.001
Hematocytopenia			
Leukopenia	2.30	1.00-5.25	0.049
Erythropenia	-	-	-
Thrombocytopenia	6.89	2.01-23.59	0.002

Adjusted for age, sex, body mass index, disease duration, hypertension, diabetes, alcoholism and smoking. CI, confidence interval; OR, odds ratio; WD, Wilson disease.

**Table 5:** Splenectomy for WD-associated cirrhotic patients with splenomegaly

	No splenectomy (n = 55)	Partial splenectomy (n = 8)	Complete splenectomy (n = 12)	P-value
Leukopenia	25 (45.45)	7 (87.50)	1 (8.33)	0.002
Erythropenia	9 (16.36)	3 (37.50)	3 (25.00)	0.337
Thrombocytopenia	25 (45.45)	5 (62.50)	0 (0)	0.006

Data are presented as n (%). WD, Wilson disease.
